# Performance evaluation of the LIOFeron®TB/LTBI IGRA for screening of paediatric LTBI and tuberculosis

**DOI:** 10.1007/s00431-025-05972-6

**Published:** 2025-01-20

**Authors:** Chiara Della Bella, Marco Antonio Motisi, Elisabetta Venturini, Sofia D’Elios, Evangelia Asvestopoulou, Agnese Maria Tamborino, Luisa Galli, Mario Milco D’Elios, Elena Chiappini

**Affiliations:** 1 Department of Molecular and Developmental Medicine, University of Siena, Siena, Italy; 2 Paediatrics resident, Department of Health Sciences, Anna Meyer Children’s University Hospital, University of Florence, Florence, Italy; 3 Infectious Diseases Unit, Department of Health Sciences, Anna Meyer Children’s University Hospital, University of Florence, Florence, Italy; 4 Department of Clinical and Experimental Medicine, University of Pisa, Pisa, Italy

**Keywords:** *Mycobacterium tuberculosis*, Tuberculosis, Diagnosis, Interferon-gamma release assay, Alanine dehydrogenase

## Abstract

Purpose: High-accuracy diagnostic screening tests for *Mycobacterium tuberculosis* (MTB) infection are required, primarily to detect patients with latent infections (LTBIs) in order to avoid their progression to active tuberculosis disease. The performance of the novel IGRA LIOFeron®TB/LTBI was evaluated in children. The originality of this test is the new MTB antigen contained (l-alanine dehydrogenase), identified as a tool to differentiate active TB from LTBI infection. Methods: From March 2022 to November 2023, a population of 90 children was enrolled and grouped into healthy, active TB or LTBI individuals, based on diagnostic guidelines. The blood of all these participants was tested with LIOFeron®TB/LTBI assay in comparison to diagnosis, as gold standard, and to the current used IGRA QuantiFERON®-TB Gold Plus. Results: The two assays demonstrated an excellent concordance of their results with patients’ diagnosis of MTB infection. The performance of LIOFeron®TB/LTBI assay in terms of accuracy of MTB infection diagnosis was high at ROC analysis (AUC = 0.997), and the test showed 100% sensitivity in LTBI detection. The QuantiFERON®-TB Gold Plus sensitivity for LTBI detection was 85.7%.

*Conclusions:* Based on the obtained results, the LIOFeron®TB/LTBI assay appears to be a promising test for TB and LTBI screening among paediatric patients.

**Table:** 

What is known: *• The detection of LTBI in children, exposed to MTB infections, followed by appropriate treatment, has a pivotal role in reducing tuberculosis burden.* *• IGRA tests are easy-to-use methods for helping large TB screening in paediatrics.*
What is new: *• The LIOFeron®TB/LTBI performance evaluation showed 100% of sensitivity in the detection of LTBI patients.* *• The LIOFeron®TB/LTBI assay might be useful for the detection of LTBI and active tuberculosis paediatric patients.*

**Supplementary Information:**

The online version contains supplementary material available at 10.1007/s00431-025-05972-6.

## Introduction

Tuberculosis (TB) is an infectious disease caused by pathogens belonging to the *Mycobacterium tuberculosis* (MTB) *complex* [1]. In 2022, the reported global number of people newly diagnosed with TB was 7.5 million, and 12% were children (0–14 years); of the global number of about 1.3 million TB deaths in 2022, children were 16% of the total [2].

It is estimated that about 1.7 billion people, including 67 million children, harbour a latent TB infection (LTBI), and it is therefore at risk of developing active TB disease [3–5]. According to WHO End TB Strategy, preventing TB infection and stopping its progression to disease are critical interventions for reducing the global TB burden. This approach is linked to early diagnosis, performed by screening tests as tuberculin skin test (TST) or interferon-gamma (IFN-γ) release assays (IGRAs), especially useful on populations at high risk for progression to active severe disease, such as children under 5 years of age [6–9]. Thus, it is necessary to improve diagnosis of both active TB and LTBI, by implementing new screening tests [10].

However, neither IGRAs nor TST can distinguish between active TB and LTBI [11, 12], and current IGRAs are more specific tests than TST, since they have been optimized with MTB *complex* specific antigens avoiding cross-reactions with Bacillus Calmette-Guérin (BCG) vaccine in immunized subjects and with the most of non-tubercular Mycobacteria (NTM) [13, 14]. IGRAs consistently show high sensitivity and specificity in children ≥ 2 years of age [15, 16], and some recent data support their use even in younger ones [17, 18].

The enzyme l-alanine dehydrogenase (Ala-DH) encoded by the *RV2780* gene of MTB may be of help in discriminating children with active TB from those with LTBI [19, 20]. Ala-DH production and activity increase in MTB under stress conditions like hypoxia, so the enzyme is one of the fundamental agents for MTB persistence during LTBI dormant stage, proposed to hold the optimal NADH/NAD^+^ ratio to prepare for regrowth through responding early to re-oxygenation [21–23]. Therefore, the detection of a specific immune response against Ala-DH could be useful to discriminate individuals with LTBI from those with active TB disease. Moreover, Ala-DH is not synthetized by BCG; thus, an Ala-DH-based IGRA should not be influenced by previous BCG immunization or infection by NTM [24].

With the aim of enhancing the detection of individuals with latent MTB infection, the novel IGRA LIOFeron®TB/LTBI test was developed by Lionex (Lionex GmbH, Braunschweig, Germany), containing the MTB Ala-DH [25].

The currently used QuantiFERON®-TB Gold Plus (QFT-GP) assay includes one test-tube containing ESAT-6, CFP-10 and TB7.7 peptides stimulating CD4^+^ T cell response and a test-tube 2 with shorter peptides from ESAT-6 and CFP-10, designed to stimulate CD8^+^ T cells too [26, 27]. The LIOFeron®TB/LTBI test differs from the QFT-GP assay because the first test tube A contains the full-length ESAT-6, CFP-10 and TB7.7, and the second test-tube B contains the highly purified recombinant Ala-DH alone.

Data about the LIOFeron®TB/LTBI test in the paediatric populations are lacking. Thus, this study was designed for evaluating the performance of the LIOFeron®TB/LTBI test, for the first time, in blood samples from children, in comparison to their TB diagnosis and to the QFT-GP assay.

## Materials and methods

### Study population

The present research was designed as a pilot single-centre prospective observational study on paediatric tuberculosis patients and controls.

Tuberculosis has no gold standard test; thus, IGRA performance was evaluated in comparison with the subject’s diagnosis in a cohort of 90 children enrolled from March 2022 to November 2023, after obtaining their parents/tutors’ informed consent for this study, in the Paediatric Infectious Diseases Unit, Department of Health Sciences, Anna Meyer Children’s University Hospital in Florence, Italy.

Inclusion criteria were as follows: children with an age < 18 years, enrolled in suspicion of MTB infection.

Exclusion criteria: presence of severe chronic comorbidity including diabetes, leukaemia or other cancer; end-stage renal disease; autoimmune disease and being on immunosuppressive therapy.

In our clinic, children were subjected to investigations for TB if they presented with suggestive clinical manifestations, including cough more than 2 weeks duration, fever, weight loss, failure to thrive or they were in recent close contacts with an infectious case and recent immigrants from/travellers to an area where TB was endemic.

The diagnosis of TB infection was determined following the current guidelines [15, 28–31]: active TB patients (aTB) were diagnosed by clinical, radiological and microbiological findings; patients with a latent TB infection (LTBI) were subjects with a previous positive TST or IGRA test recently determined in the absence of clinical symptoms nor radiological abnormalities at a chest X-ray. Information regarding medical history was obtained from each child’s parents or tutors or from available medical documentation and recorded into the study database. Healthy children who had negative tests for TB and patients with NTM infection were enrolled as control subjects. Healthy controls (HCs) were paediatric subjects with no symptoms, no alterations at clinical examination and with at least one recent negative TST/IGRA result (e.g. subjects who underwent TB screening for recent immigration); patients with NTM infection were diagnosed by the presence of lymphadenitis and a NTM-positive culture from the bioptic specimen.

### IGRA testing

The principle of the IGRA method is the IFN-γ detection by enzyme-linked immunosorbent assay (ELISA) into plasma samples derived from whole blood samples subjected to specific MTB antigen stimulation. The amount of IFN-γ released is an index of the cell-mediated immune response to MTB infection. Eight millilitres of blood, drawn by venipuncture from each child, was collected in lithium-heparin and then, following the manufacturer’s instructions, the two IGRAs, LIOFeron®TB/LTBI assay [32] and QFT-GP test [33], were simultaneously performed within 16 h. In some children, the two tests have been performed with a few days break to avoid sampling too much blood in younger patients.

### LIOFeron®TB/LTBI assay

The collected whole-blood sample was pipetted 1 ml each into negative, positive and TB antigen stimulation tubes A and B, provided in the kit. The tubes were gently mixed, in order to dissolve in the blood the antigens on tube walls, and placed into a 37 °C incubator overnight for about 16–20 h. Plasma was carefully removed and frozen at − 20 °C. The amount of released cytokine IFN-γ into the plasma samples was quantified in a second step by human IFN-γ ELISA. Samples, IFN-γ standards (4.0 IU/ml, 1.0 IU/ml, 0.25 IU/ml) and the blank were diluted in an incubation buffer and then seeded into the plate wells, pre-coated with anti-IFN-γ antibody. Detection antibody, conjugate solution and, finally, the substrate solution were in sequence added, alternating with washes and incubations. The obtained intensity of the colour reaction was measured as optical density (OD) and quantified at a 450 nm wavelength with a spectrophotometer (Multiskan Go, Thermo Scientific, Massachusetts, USA). ELISA data were analysed with the validated LIOferon® software (free downloaded from the Lionex web page) to obtain the automatic generation of the standard curve, the interpolation of samples data to define their IFN-γ concentration (IU/ml), the blank subtraction and the 25% of negative value calculation. Results were reported as positive, negative and indeterminate upon quantitative values (as detailed in Table S1, in Supplementary Informations). Reagents of the LIOFeron®TB/LTBI kit were kind gifts of Lionex GmbH.

### QuantiFERON®-TB Gold Plus assay

According to the manufacturer’s guidelines, the four blood stimulation tubes Nil, TB-1, TB-2 and mitogen were filled with 1 ml of the child’s blood. After shaking, tubes were then incubated for 16–20 h at 37 °C. After incubation, plasma was harvested and stored at − 20 °C. The plasmas were then processed by the supplied IFN-γ ELISA assay for the cytokine amount detection. The validated QuantiFERON® software, available online on the manufacturer’s website, was used to calculate results after photometric analysis at 450 nm. The results interpretation is summarised in Table S1.

### Statistical analysis

Data analyses were carried out with the SPSS Statistics software, version 29.0.0.0 (SPSS Italia SRL, Bologna, Italy). Descriptive statistic was used for the calculation of absolute frequencies and percentages of qualitative data, as well as for mean and standard deviation of quantitative data. IGRA sensitivity, specificity, positive predictive value (PPV), negative predictive value (NPV), a positive likelihood ratio (PLR) and a negative likelihood ratio (NLR) were computed on contingency tables. The agreement between IGRAs results with patient diagnosis was assessed by calculating the percentage of concordant results and by computing the Cohen’s Kappa coefficient [34]. Continuous variables were analysed for their normality of distribution by the Shapiro–Wilk test. Test performance was evaluated for each IGRA by a receiving operating characteristic (ROC) curve. The area under the curve (AUC) is a parameter to assess the test accuracy [35]. Youden’s index was applied to evaluate the best cut-off in our experimental data distributions [36]. DeLong’s test was used to assess the statistical significance of the difference between the AUCs (*p* < 0.05) [37].

## Patients’ medical reports

A total of 90 children were included: 53 HC, 15 aTB patients, 16 LTBI and 6 NTM patients. The male/female ratio was 58/32, with a median age of 7 years and 3 months; 29/90 children were younger than 5 years. More specific demographic features of patients are described in Table 1. Clinical characteristics of children with active TB are described in Table 2.

**Table 1:** Selected study participants and their demographic data. Grouping criteria of selected study participants and their demographic characteristics. Age is expressed as (years, months) and calculated as median and interquartile range (IQR)

		aTB	LTBI	HC	NTM infection	Total
*N*		15	16	53	6	90
Age	Median	6y, 5 m	9y, 9 m	7y, 6 m	2y, 6 m	7y, 3 m
	IQR	4–12 y, 5 m	6 y, 4 m–12 y, 3 m	4–10 y	1 y, 7 m–6 y, 11 m	3 y, 11 m–10 y, 10 m
Gender (%)	Males	10 (66.7)	10 (62.5)	37 (69.8)	1 (16.7)	58
	Females	5 (33.3)	6 (37.5)	16 (30.2)	5 (83.3)	32
Ethnicity (%)	Europe	9 (60.0)	4 (25.0)	20 (37.7)	5 (83.3)	38
	Africa	4 (26.7)	3 (18.7)	12 (22.5)	1 (16.7)	20
	South America	1 (6.7)	5 (31.3)	4 (7.5)	0 (0)	10
	Asia	1 (6.7)	4 (25.0)	17 (32.3)	0 (0)	22
BCG status (%)	Vaccinated	0 (0.0)	8 50.0)	31 (58.5)	0 (0)	39
	Unvaccinated	6 (40.0)	5 (31.2)	6 (11.3)	6 (100)	23
	Unknown	9 (60.0)	3 (18.8)	16 (30,2)	0 (0)	28
TST result (%)	Positive	9 (60.0)	9 (56.2)	0 (0)	3 (50)	21
	Negative	1 (6.7)	2 (12.5)	15 (28.3)	3 (50)	21
	Not performed	5 (44.0)	5 (31.39)	38 (71.7)	0 (0)	48
QFT-GP result (%)	Positive	13 (92.8)	12 (85.7)	0 (0)	0 (0)	25
	Negative	1 (7.2)	2 (14.3)	52 (98.1)	6 (100)	61
	Indeterminate	0 (0.0)	0 (0.0)	1 (1.9)	0 (0)	1

**Table 2:** Clinical characteristics of children with active TB. Of the two children with extrapulmonary disease, one had lymph node involvement and the other one had osteoarticular infection. *PCR* polymerase chain reaction, *AFB* acid-fast bacilli

	Children with aTB (*N* = 15)	
Sex (%)	Male	10 (66.7)
	Female	5 (33.3)
Age (%)	0–1 years old	1 (6.7)
	1–2 years old	1 (6.7)
	3–4 years old	3 (15.0)
	5–13 years old	4 (26.7)
	> 13 years old	6 (44.9)
Nativity (%)	Born in Italy from Italian parents	7 (46.7)
	Born in Italy from foreign parents	2 (13.3)
	Born outside Italy	6 (40.0)
Continent of origin (%)	Asia	1 (6.7)
	South-central America	1 (6.7)
	North Africa	1 (6.7)
	South Central Africa	1 (6.7)
	Sub-Saharan Africa	2 (13.4)
	Italy	7 (46.7)
	Europe	2 (13.4)
Reason for investigation (%)	Contact with patient with suspected or confirmed TB	5 (33.3)
	Adoption or immigration screening	0 (0.0)
	Symptoms	10 (66.7)
Clinical manifestations (%)	Pulmonary, non-miliary	8 (53.3)
	Pulmonary, miliary	4 (26.6)
	Pleural effusion	1 (6.7)
	Extrapulmonary TB	2 (13.4)
PCR testing for mycobacteria (%)	Positive	8 (53.2)
	Negative	7 (46.8)
Microscope detection for AFB (%)	Positive	2 (6.7)
	Negative	13 (92.3)
Mycobacterial culture (%)	Positive	7 (46.7)
	Negative	8 (53.3)

Four patients were excluded from the following statistical computing. Three patients with MTB infection (one with active TB and two with LTBI) were excluded from comparative evaluation between the two IGRAs because only TST, with positive result, was performed and no QFT-GP. These three patients indeed were analysed for their concordance between LIOFeron®TB/LTBI assay and diagnosis of MTB infection; they also were included into LIOFeron®TB/LTBI ROC analysis. One HC was excluded from the statistical analysis due to indeterminate test result in both assays.

## Results

### High agreement between LIOFeron®TB/LTBI assay result and subject’s diagnosis

Both IGRAs classified all (52/52; 100% specificity) the healthy subjects as negative. 30/31 MTB infected patients (aTB + LTBI) tested with LIOFeron®TB/LTBI assay resulted as true positive (96.8% sensitivity); only one aTB patient was a false negative. Regarding QFT-GP results, 25/28 (89.3% sensitivity) patients with MTB infection (13/14; 92.8% with active TB and 12/14; 85.7% with LTBI) had a positive result and 3/28 (10.7%) were wrongly identified as negative (1/14 with active TB and 2/14 with LTBI). The LIOFeron®TB/LTBI assay showed a PPV = 1, a NPV = 0.98, a PLR > 10 and a NLR = 0.032. QFT-GP showed a PPV = 1, a NPV = 0.94, a PLR > 10 and a NLR = 0.107.

None of the two assays resulted positive in NTM infected patients, confirming no cross reaction with MTB testing. All three subjects with false negative QFT-GP results were children under 5 years of age and the LIOFeron®TB/LTBI assay was positive in all of them. In these cases, diagnosis of MTB infection was possible because of TST positivity or chest X-ray alterations. The age of the aTB patient resulted as LIOFeron®TB/LTBI false negative was 6 years, 5 months, and it was rightly diagnosed by QFT-GP. IGRA results in all patients and in 29 children < 5 years old are reported in Tables 3 and 4, respectively.

**Table 3:** Agreement of both IGRAs results with diagnosis in all patients

		LIOFeron®-TB/LTBI [*N* (%)]			QuantiFERON®-TB Gold Plus [*N* (%)]		
Diagnosis		Positive	Negative	Total	Positive	Negative	Total
HC		0 (0%)	52 (100%)	52	0 (0%)	52 (100%)	52
MTB infected	aTB	14 (93.3%)	1 (6.7%)	15	13 (92.8%)	1 (7.2%)	14
	LTBI	16 (100%)	0 (0%)	16	12 (85.7%)	2 (14.3%)	14
NTM		0 (0%)	6 (100%)	6	0 (0%)	6 (100%)	6
Total		30	59	89	23	60	86

**Table 4:** Agreement of both IGRAs results with diagnosis in patients under 5 years of age

		LIOFeron-TB/LTBI [*N* (%)]			QuantiFERON- TB Gold Plus [*N* (%)]		
Diagnosis		Positive	Negative	Total	Positive	Negative	Total
HC		0 (0%)	16 (100%)	16	0 (0%)	16 (100%)	16
MTB infected	aTB	5 (100%)	0 (0%)	5	4 (80.0%)	1 (20.0%)	5
	LTBI	3 (100%)	0 (0%)	3	1 (33.3%)	2 (66.7%)	3
NTM		0 (0%)	5 (100%)	5	0 (0%)	5 (100%)	5
Total		8	21	29	5	24	29

Considering BCG immunization, 28/90 (31.1%) patients’ vaccination status was unknown. Of the remaining subjects, 39/62 (62.9%) received one dose of BCG at birth and 23/62 (37.1%) were not vaccinated. Of the three patients with discordance between the two IGRAs, two of them were vaccinated. Cohen’s *k* coefficient was computed to evaluate the accordance between the diagnosis and the IGRA test result (positive/negative). A coefficient *k* = 0.91 was obtained for QFT-GP, a *k* = 0.97 for LIOFeron®TB/LTBI, reaching for both assays an excellent level of concordance of their results with patients’ diagnosis of MTB infection.

### Performance of LIOFeron®TB/LTBI in terms of test accuracy

In order to define the diagnostic accuracy of the LIOFeron®TB/LTBI IGRA, a ROC analysis was applied. The performance analysis of the two IGRAs using ROC curve was conducted by combining the effect of the two test tubes (TB-1 + TB-2) and (TB-A + TB-B). ROC analysis demonstrated both IGRAs as highly accurate assays.

The IGRAs ROC curve analysis at the manufacturer recommended cut-off defined for the LIOFeron®TB/LTBI assay had a sensitivity of 96.8%, 100% and 100% in discriminating between HC vs MTB infected, HC vs aTB and HC vs LTBI, respectively. The LIOFeron®TB/LTBI assay AUC in the plot of HC vs MTB infected was 0.997 (CI 95%: 0.991–1.003), the AUC of the HC vs aTB ROC curve was 0.995 (CI 95%: 0.983–1.007) and the AUC in the HC vs LTBI ROC graphic was 0.999 (CI 95%: 0.997–1.002). The sensitivity of QFT-GP at the manufacturer recommended cut-off in the ROC curve was 89.3%, 92.9% and 85.7% in discriminating between HC vs MTB infected, HC vs aTB and HC vs LTBI, respectively. The accuracy of QFT-GP in the HC vs MTB infected test, HC vs aTB test and HC vs LTBI test was defined, in order, as AUC = 0.922 (CI 95%: 0.837–1.007), AUC = 0.952 (CI 95%: 0.860–1.044) and AUC = 0.891 (CI 95%: 0.751–1.032).

**Fig. 1:**
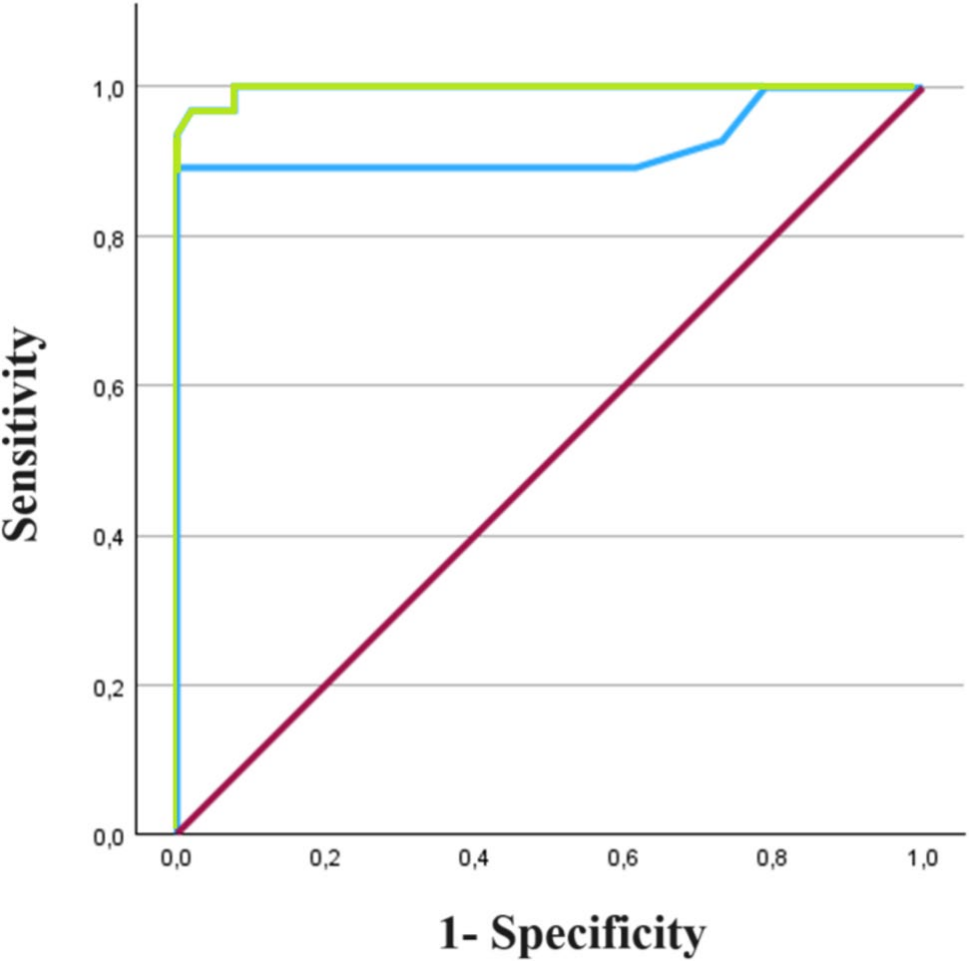
ROC curve showing the performance of LIOFeron®TB/LTBI IGRA compared to the one of QuantiFERON®-TB Gold Plus IGRA in MTB infection detection. The green line depicts the AUC for LIOFeron®TB/LTBI IGRA (0.997) and the blue one outlines the AUC (0.922) for the QFT-GP assay

The two IGRAs’ performance evaluation was calculated by the pairwise comparison of all the AUCs; the comparison between LIOFeron®TB/LTBI assay and QFT-GP AUCs for HC vs MTB infected resulted significantly different (*p* = 0.045). Results are summarized in Table 5 and Fig. 1.

**Table 5:** Test performance by ROC analysis at the cut-off recommended by the company (0.35 IU/ml)

		QuantiFERON®-TB Gold Plus (TB-1 + TB-2)	LIOFeron®TB/LTBI (TB-A + TB-B)
Sensitivity	HC vs MTB infected	89.3%	96.8%
	HC vs aTB	92.9%	100.0%
	HC vs LTBI	85.7%	100.0%
AUC (CI 95%)	HC vs MTB infected	0.922 (0.837–1.007)	0.997 (0.991–1.003)
		*p* = 0.045	
	HC vs aTB	0.952 (0.860–1.044)	0.995 (0.983–1.007)
		*p* = 0.315	
	HC vs LTBI	0.891 (0.751–1.032)	0.999 (0.997–1.002)
		*p* = 0.069

Furthermore, the performance evaluations of both IGRAs were also calculated for the speculated best cut-off capable of discriminating in the best way between HC and MTB infected persons, but also between HC and aTB patients and between HC and LTBI subjects. In particular, the LIOFeron®TB/LTBI ROC curve analysis indicated the same high sensitivity of this test at both manufacturer recommended cut-off and at the Youden’s best cut-off (96.8% in HC vs MTB infected; 100% in HC vs aTB and the same for HC vs LTBI) as highlighted in Table S2.

## Discussion

According to the updated literature, many studies have been performed on IGRA’s accuracy evaluation in children populations, gaining a variety of different results [38–46]. Hirabayashi et al. [47] performed a recent systematic review on 47 selected studies about the accuracy of IGRAs on children populations, demonstrating that the sensitivity and specificity of the IGRAs in children were moderate and high, respectively, confirming the IGRAs as useful tools for detecting MTB infection in children. On August 2024, the World Health Organization (WHO) has published a public call for data in order to update the WHO guidance on IGRAs for detection of MTB infection. The IGRAs that are currently recommended by the WHO for diagnosis of MTB infection include the QuantiFERON-TB Gold Plus (QFT-GP, Qiagen), the T-SPOT.TB (Oxford Immunotec), and the Wantai TB-IGRA (Beijing Wantai Biological Pharmacy Enterprise) [48]. No specific articles focusing on children populations are available regarding the Wantai TB-IGRA; a 2023 systematic review from Ortiz-Brizuela et al. [49] concluded that QFT-Plus and the Wantai TB-IGRA have very similar sensitivity and specificity as WHO-approved IGRAs in adults. A systematic review and meta-analysis on QFT-Plus, published in 2019, showed greater sensitivity of QFT-Plus in children with active TB disease, but also in children with recent MTB exposure [50]. Moreover, in a recent prospective cross-sectional study, Buonsenso et al. demonstrated that QFT-Plus assay had good sensitivity for active TB and was particularly useful for the evaluation of children with suspected LTBI, but there was no statistical significance to conclude that QFT-Plus was able to distinguish active TB from LTBI [51]. This is the first manuscript describing the performance of the LIOFeron®TB/LTBI test in the paediatric age. The clinical interest towards this novel IGRA relies primarily in its higher sensitivity for LTBI when compared to reference IGRA, along with good sensitivity also for the active TB and high specificity demonstrated in the 2020 study [20]. The purpose of this research was to evaluate the performance of this test in children with MTB infection and in healthy subjects, comparing the results with those obtained with the QFT-GP.

Our findings, interpreting the results at the cut-off recommended by the company (0.35 IU/ml), demonstrated LIOFeron®TB/LTBI to be capable of diagnosing MTB infection with a high sensitivity (96.8%) in both aTB and LTBI groups, with no false positive among HC or NTM. The sensitivity of QFT-GP was 89.3% (92.8% for aTB and 85.7% for LTBI patients). We found a 7.5% increase in detection of real positive MTB patients showed by the LIOFeron®TB/LTBI assay compared to QFT-GP. The ROC analysis performed to define the two IGRAs’ performance in identifying LTBI cases, using the manufacturer’s cut-off, showed 100% of sensitivity in LIOFeron®TB/LTBI and 85.7% in QFT-GP assay. The results obtained in this study could be an important advantage of the LIOFeron®TB/LTBI test in the detection of LTBI patients that could be a matter of further investigations. Thus, these results obtained in children together with the ones obtained so far on adult cohorts [20] and as suggested by Gong et al. [52], LIOFeron®TB/LTBI would be a promising test for TB and LTBI screening among paediatric patients. The detection of LTBI in children, exposed to MTB infections, followed by appropriate treatment, has a pivotal role in reducing TB burden. All the IGRAs are considered to be very useful in evaluating LTBI [53]. Thus, the LIOFeron®TB/LTBI IGRA might represent an important IGRA available as tool for healthcare interventions concerning TB prevention by early diagnosis that will reduce the risk of progression from LTBI to active disease.

TB is challenging to diagnose, especially in children, because there is no gold standard test; IGRA results are associated on memory T cell response; thus active TB is used as a surrogate to measure the sensitivity of these assays. Moreover, IGRAs are unable to distinguish between TB infection and active TB disease and can give positive results in case of some NTM infections.

This study has some limitations, and wider research works are needed to validate the accuracy of the LIOFeron®TB/LTBI assay in children. Given that this investigation represents a pilot single-centre study, future multi-centre research activities dealing with LIOFeron®TB/LTBI assay in paediatrics would be very important for a better definition of the test. In addition, a long-term follow-up (1–2 years) of children with negative test results could be also important to underline eventual progress to aTB.

In conclusion, the results obtained so far indicate that LIOFeron®TB/LTBI displays high accuracy in diagnosing MTB infection/TB disease as well as LTBI detection in paediatrics. The features of LIOFeron®TB/LTBI test can lead it to have a role in WHO strategies for ending TB.

## Supplementary Information

Below is the link to the electronic supplementary material.

Supplementary file1 (DOCX 15 KB)

## Data Availability

Information regarding the diagnosis of MTB infection, demographic data, prior TB exposure, BCG vaccination and past medical history of the enrolled children were obtained from each child's parents or tutors or from available medical documentation and recorded into the Anna Meyer Children’s University Hospital database, in care of Prof. E. Chiappini. Raw data of IGRA tests have been recorder in University of Siena database, in the custody of Prof. MM D'Elios. Both databases are available upon request.

## Abbreviations

aTB: Active TB
AUC: Area under the curve
BCG: Bacillus Calmette-Guérin
ELISA: Enzyme-linked immunosorbent assay
HC: Healthy control
IFN-γ: Interferon-gamma
IGRA: Interferon-gamma release assays
Ala-DH: L-Alanine dehydrogenase
LTBI: Latent tuberculosis infection
MTB: *Mycobacterium tuberculosis*
NTM: Non-tubercular Mycobacteria
QFT-GP: QuantiFERON®-TB Gold Plus
ROC: Receiving operating characteristic
TB: Tuberculosis
TST: Tuberculin skin test
