# Supplementary material for: Performance evaluation of the LIOFeron®TB/LTBI IGRA for screening of paediatric LTBI and tuberculosis
Source: Eur J Pediatr. 2025 Jan 20;184(2):147. doi: 10.1007/s00431-025-05972-6 (PMC11753305; doi:10.1007/s00431-025-05972-6)
Supplement: Supplementary file 1 — Supplementary file1 (DOCX 15 KB) [file 431_2025_5972_MOESM1_ESM.docx]

Supplementary Information:

**Table S1**. IGRA results interpretation.

| **Negative control (IU/ml)** | **First antigen tube minus negative control (IU/ml)** | **Second antigen tube minus negative control (IU/ml)** | **Positive control minus negative control (IU/ml)** | **Test result** |
| --- | --- | --- | --- | --- |
| ≤ 8 | < 0.35 | < 0.35 | ≥ 0.50 | NEGATIVE |
| ≤ 8 | ≥0.35 and <25% of negative control value | ≥0.35 and <25% of negative control value | ≥ 0.50 | NEGATIVE |
| ≤ 8 | < 0.35 | ≥0.35 and <25% of negative control value | ≥ 0.50 | NEGATIVE |
| ≤ 8 | ≥0.35 and <25% of negative control value | < 0.35 | ≥ 0.50 | NEGATIVE |
| ≤ 8 | ≥0.35 and ≥25% of negative control value | Not relevant | Not relevant | POSITIVE |
| ≤ 8 | Not relevant | ≥0.35 and ≥25% of negative control value | Not relevant | POSITIVE |
| ≤ 8 | < 0.35 | < 0.35 | < 0.50 | INDETERMINATE |
| ≤ 8 | ≥0.35 and <25% of negative control value | ≥0.35 and <25% of negative control value | < 0.50 | INDETERMINATE |
| ≤ 8 | < 0.35 | ≥0.35 and <25% of negative control value | < 0.50 | INDETERMINATE |
| ≤ 8 | ≥0.35 and <25% of negative control value | < 0.35 | < 0.50 | INDETERMINATE |
| > 8 | Not relevant | Not relevant | Not relevant | INDETERMINATE |

**Table S2**. Test accuracy by ROC analysis at the best cutoff.

|  | Diagnosis of MTB infection | Sensitivity | Specificity | Best cutoff (IU/ml)  (Youden’s index) |
| --- | --- | --- | --- | --- |
| LIOFeron^®^TB/LTBI  (TB-A + TB-B) | HC vs MTB infected | **96.8%** | 98.1% | 0.70 |
|  | HC vs aTB | **100.0%** | 92.3% | 0.29 |
|  | HC vs LTBI | **100.0**% | 98.1% | 0.70 |
